# Myocardial injury in hospitalized patients with COVID-19 infection—Risk factors and outcomes

**DOI:** 10.1371/journal.pone.0247800

**Published:** 2021-02-26

**Authors:** Orly Efros, Noam Barda, Eshcar Meisel, Avshalom Leibowitz, Alexander Fardman, Galia Rahav, Robert Klempfner, Ehud Grossman

**Affiliations:** 1 Sheba Medical Center, Tel-Hashomer, Ramat Gan, Israel; 2 Sackler Faculty of Medicine, Tel-Aviv University, Tel-Aviv, Israel; 3 Clalit Research Institute, Clalit Health Services, Tel-Aviv, Israel; Ohio State University, UNITED STATES

## Abstract

Myocardial injury in hospitalized patients is associated with poor prognosis. This study aimed to evaluate risk factors for myocardial injury in hospitalized patients with coronavirus disease 2019 (COVID-19) and its prognostic value. We retrieved all consecutive patients who were hospitalized in internal medicine departments in a tertiary medical center from February 9^th^, 2020 to August 28^th^ with a diagnosis of COVID-19. A total of 559 adult patients were hospitalized in the Sheba Medical Center with a diagnosis of COVID-19, 320 (57.24%) of whom were tested for troponin levels within 24-hours of admission, and 91 (28.44%) had elevated levels. Predictors for elevated troponin levels were age (odds ratio [OR], 1.04; 95% confidence interval [CI], 1.01–1.06), female sex (OR, 3.03; 95% CI 1.54–6.25), low systolic blood pressure (OR, 5.91; 95% CI 2.42–14.44) and increased creatinine level (OR, 2.88; 95% CI 1.44–5.73). The risk for death (hazard ratio [HR] 4.32, 95% CI 2.08–8.99) and a composite outcome of invasive ventilation support and death (HR 1.96, 95% CI 1.15–3.37) was significantly higher among patients who had elevated troponin levels. In conclusion, in hospitalized patients with COVID-19, elevated troponin levels are associated with poor prognosis. Hence, troponin levels may be used as an additional tool for risk stratification and a decision guide in patients hospitalized with COVID-19.

## Introduction

The coronavirus disease 2019 (COVID‐19) pandemic is a global health concern. Its main manifestation is pulmonary inflammation with potentially life-threatening complications, such as pneumonia and acute respiratory distress syndrome [1–3].

Myocardial injury, as heralded by elevated troponin levels, was previously observed in various medical conditions, including pneumonia [4–6], and was found to be correlated with adverse outcomes such as cardiac complications and death [6–8].

To date, several studies have described myocardial injury in patients diagnosed with COVID-19 [1, 9–17]. According to these studies, COVID-19 complications such as acute respiratory distress syndrome, acute kidney injury were more common in patients with myocardial injury. Furthermore, myocardial injury was shown to be independently associated with an increased risk of mortality. Patients with cardiovascular disease were more likely to experience a myocardial injury. Nonetheless, myocardial injury was associated with increased mortality regardless of the presence of cardiovascular disease or risk factors [11, 15]. In a recent single-center retrospective study, an elevated troponin level upon admission was associated with a higher in-hospital mortality, and was more common among older patients, with more comorbidities [16]. In a retrospective study from several Italian institutions, COVID-19 patients with and without chronic coronary syndrome (CCS) were classified and compared accordingly. Interestingly however, troponin elevation was found to have a prognostic value in non-CCS patients, while in CCS patients it was not found to predict poor outcome. The authors suggested that myocardial injury may be a bystander in CCS patients with COVID-19, while in patients without CCS, myocardial injury has a significant role and thus was found to have a prognostic value [17].

Our study aimed to further evaluate the incidence of myocardial injury in COVID-19 hospitalized patients, to characterize risk factors for myocardial injury among these patients, to determine its prognostic significance.

## Methods

### Study design and population

This is a retrospective cohort study. Data were obtained from the electronic medical records of the Sheba Medical Center, the largest tertiary medical center operating in Israel. We retrieved all consecutive patients who were hospitalized from February 9^th^, 2020, to August 28^th^, 2020, with a diagnosis of COVID-19 and a reverse-transcription polymerase chain reaction confirmed SARS-CoV-2 infection. All patients included were 18 years old or older at the time of diagnosis and had at least one high sensitivity troponin (hs-TnT) measurement within 24 hours after admission. If there was more than one hs-TnT measurement into the first 24-hours, the highest level was chosen.

### Data collection and outcomes

For each patient, baseline demographic characteristics and clinical information were extracted from the medical records. Clinical data included medical comorbidities, home medications, vital signs on admission (body temperature, systolic blood pressure, and oxygen saturation), and laboratory tests that were taken within 24-hours from admission (normal laboratory reference ranges are available in S1 Table). Comorbidities were defined based on the International Classification of Disease, 10^th^ Revision codes and included heart failure, ischemic heart disease, chronic kidney disease, malignancy, chronic obstructive pulmonary disease, diabetes mellitus, hypertension, cerebrovascular accident, and dyslipidemia. When death occurred outside the hospital, mortality dates were obtained from governmental mortality records.

The primary outcome was mortality. A secondary outcome was defined as a composite outcome of invasive ventilation and mortality. We also examined other outcomes, including the death within 60 days of hospitalization; intubation within 30 days from admission, which includes only the patients who were intubated during the first hospitalization with COVID-19 in The Sheba Medical Center; length of hospital stay; hypoxemia during hospitalization, defined as a measurement of oxygen saturation <90% by a pulse oximeter; acute kidney injury (AKI), defined according to the Kidney Disease: Improving Global Outcomes (KDIGO) definition as a ratio of more than 1.5 between the maximal creatinine level to its minimal value in less than seven days [18]. Myocardial injury was defined by The Fourth Universal Definition of Myocardial Infarction as an elevation of troponin levels above the 99^th^ percentile [19].

To better characterize COVID-19 patients with increased troponin, we reviewed their medical records, and examined their electrocardiogram (ECG) at presentation and compared them to previous ECG exams, coronary catheterization during hospitalization, and diagnostic imaging for pulmonary embolism (including chest computed tomography angiography (CTA) and lower limb venous Doppler ultrasound (US)).

The study was conducted and reported in accordance with the Strengthening the Reporting of Observational Studies in Epidemiology (STROBE) reporting guidelines [20].

### Statistical analysis

Baseline clinical data were compared between patients with at least one troponin measurement within 24-hours from admission and patients without troponin measurement and between patients with elevated troponin and non-elevated troponin. Continuous variables were compared using the Mann–Whitney–Wilcoxon test, and categorical variables were compared using the Chi-square test.

Logistic regression was applied to identify variables that are best associated with an elevated troponin level following COVID-19 diagnosis. Covariates for the multivariable models were pre-specified based on clinical relevance.

Outcomes of mortality, invasive ventilatory support, length of hospital stay, and AKI were analyzed in the following manner. The crude association between troponin levels and mortality, and between troponin levels and the composite outcome of invasive ventilation and mortality was modeled using Kaplan Meier curves and compared using the Log Rank test. A Cox proportional hazard regression analysis was performed to adjust for age, sex, background comorbidities (atrial fibrillation, ischemic heart disease, heart failure, hypertension, diabetes mellitus, chronic kidney disease), chronic medications (angiotensin-converting enzyme, angiotensin II receptor blockers, beta-blockers, and HMG CoA reductase inhibitors), low systolic blood pressure at admission (defined as < 90 millimeters of mercury [mmHg]), and maximal creatinine level as measured up to 24-hours from admission.

Data analyses were performed using the R programming language (R Development Core Team, version 3.6.2, Vienna, Austria).

### Ethics

The study was approved by the institutional review board (IRB) of Sheba Medical Center. Informed consent was waived by the IRB committee.

## Results

### Baseline characteristics

From February 9^th^, 2020 to August 28^th^, 2020, a total of 559 patients over the age of eighteen were hospitalized with a diagnosis of COVID-19 at the Sheba Medical Center. Among these patients, 320 (57.24%) were tested for troponin levels in the emergency room and up to 24 hours from admission. Among patients tested for troponin, 91 (28.44%) were found to have an elevated level. A visualized presentation of troponin levels is available in S1 and S2 Figs, and according to sex in S3–S6 Figs.

Patients who were tested for troponin were more likely to be male, were older, and had more underlying diseases than those who were not tested (S2 Table).

Characteristics of patients who had troponin levels measured are presented in Table 1, stratified according to troponin levels. Those who had elevated troponin levels were more likely to be female, were significantly older, and tended to have more underlying diseases. They were also more likely to be prescribed with beta-blockers and HMG- CoA-reductase inhibitors. Patients with elevated troponin had lower admission systolic blood pressure, a higher creatinine level, and lower hemoglobin and albumin levels. Characteristics of patients who had elevated troponin, stratified according to sex, are presented in S3 Table. Female patients with elevated troponin were significantly older, without more comorbidities except for diabetes mellitus.

**Table 1:** Characteristics of patients at baseline.

	Non-Elevated Troponin	Elevated Troponin	p-value	Missing values (%)
**N**	229	91		
**Sex = Male (%)**	155 (67.7)	50 (54.9)	0.044	0
**Age- years (median [IQR** ^**a**^**])**	59.83 [49.65, 71.19]	73.31 [61.33, 82.25]	<0.001	0
**No cardiac comorbidity** ^**b**^**- no. (%)**	192 (83.8)	52 (57.1)	<0.001	0
**Atrial Fibrillation- no. (%)**	17 (7.4)	19 (20.9)	0.001	0
**Heart Failure- no. (%)**	10 (4.4)	17 (18.7)	<0.001	0
**Ischemic Heart Disease- no. (%)**	21 (9.2)	22 (24.2)	0.001	0
**Chronic Kidney Disease- no. (%)**	19 (8.3)	20 (22.0)	0.001	0
**Hypertension- no. (%)**	84 (36.7)	54 (59.3)	<0.001	0
**Cerebrovascular Accident- no. (%)**	17 (7.4)	12 (13.2)	0.16	0
**Chronic Obstructive Pulmonary Disease- no. (%)**	4 (1.7)	7 (7.7)	0.022	0
**Diabetes Mellitus- no. (%)**	53 (23.1)	30 (33.0)	0.095	0
**Dyslipidemia- no. (%)**	71 (31.0)	34 (37.4)	0.337	0
**Malignancy- no. (%)**	24 (10.5)	15 (16.5)	0.197	0
**ACEI** ^**c**^ **/ ARB** ^**d**^ **Therapy- no. (%)**	21 (9.2)	15 (16.5)	0.095	0
**BB** ^e^ **Therapy- no. (%)**	42 (18.3)	39 (42.9)	<0.001	0
**HMG CoA reductase inhibitors Therapy- no. (%)**	68 (29.7)	40 (44.0)	0.021	0
**Temperature- celsius (median [IQR** ^**a**^**])**	38.00 [37.30, 38.60]	38.10 [37.20, 38.70]	0.899	0
**SBP** ^**f**^^,^ ^**g**^ **–mmHg**^**h**^ **(median [IQR** ^**a**^**])**	111.00 [101.00, 122.00]	105.00 [85.00, 122.00]	0.004	0
**Creatinine** ^**g**^**- mg/dl (median [IQR** ^**a**^**])**	0.89 [0.71, 1.12]	1.02 [0.80, 1.39]	<0.001	0
**Albumin** ^**g**^ **- g/dl (median [IQR** ^**a**^**])**	3.80 [3.60, 4.10]	3.50 [3.20, 3.80]	<0.001	2.2
**Hemoglobin** ^**g**^ **- g/dl (median [IQR** ^**a**^**])**	13.65 [12.45, 14.60]	12.70 [11.65, 14.20]	0.003	0
**Lymphocytes** ^**g**^ **K/μl (median [IQR** ^**a**^**])**	1.02 [0.74, 1.41]	0.87 [0.60, 1.28]	0.028	0
**Platelets** ^**g**^ **- K/μl (median [IQR** ^**a**^**])**	184.00 [141.00, 234.00]	198.00 [152.00, 264.50]	0.04	0
**CRP** ^**h**^^,^ ^**g**^**- mg/l (median [IQR** ^**a**^**])**	64.24 [26.32, 128.92]	92.67 [37.67, 168.22]	0.05	1.2

^a^ interquartile range

^b^ Including atrial fibrillation, heart failure or ischemic heart disease

^c^ angiotensin-converting-enzyme inhibitors

^d^ angiotensin II receptor blocker

^e^ beta-blockers

^f^ systolic blood pressure

^g^ Measured within 24-hours from admission.

^h^ millimeter of mercury

^i^ c-reactive protein.

### Predictors for elevated troponin in patients with COVID-19 Infection

In a multivariable analysis, age (OR, 1.04; 95% confidence interval [CI], 1.01–1.06), low systolic blood pressure at admission (OR, 5.91; 95% CI, 2.42–14.44), and elevated creatinine level at admission (OR, 2.88; 95% CI, 1.44–5.73) were found to be significantly associated with an elevated troponin. Male sex was significantly negatively associated with elevated troponin (OR, 0.33; 95%, CI 0.16–0.65). Other background comorbidities and chronic medications were not found to be significantly associated with elevated troponin levels. The complete data regarding predictors for elevated troponin in COVID-19 patients are presented in Table 2.

**Table 2:** Predictors for elevated troponin in presence of COVID-19 diagnosis.

	Adj. OR ^a^ (95% CI ^b^)	p-value
**Age**	1.04 (1.01,1.06)	0.002
**Sex = Male**	0.33 (0.16,0.65)	0.002
**SBP** ^**c**^ **<90 mmHg**	5.91 (2.42,14.44)	< 0.001
**Creatinine** ^**c**^	2.88 (1.44,5.73)	0.003
**Atrial Fibrillation**	1.06 (0.39,2.88)	0.913
**Ischemic Heart Disease**	1.81 (0.72,4.55)	0.205
**Heart Failure**	1.98 (0.69,5.72)	0.207
**Chronic Kidney Disease**	0.86 (0.31,2.4)	0.77
**Diabetes Mellitus**	0.93 (0.44,1.95)	0.849
**Cerebrovascular Accident**	1.01 (0.35,2.88)	0.986
**Hypertension**	0.97 (0.48,1.96)	0.931
**Beta Blockers Therapy**	1.24 (0.58,2.63)	0.582
**ACEI** ^**d**^ **/ ARB** ^**e**^ **Therapy**	1.45 (0.54,3.85)	0.458
**HMG CoA reductase inhibitors**	1.03 (0.5,2.11)	0.93
**C-Reactive Protein** ^**c**^	1 (1,1.01)	0.354
**Lymphopenia** ^**c**^	1.4 (0.73,2.68)	0.306

^a^ adjusted odds ratio

^b^ confidence interval

^c^ Measured within 24-hours from admission

^d^ angiotensin-converting-enzyme inhibitors

^e^ angiotensin II receptor blocker

### Patient outcomes

Prior to adjustment, patients with elevated troponin level were more likely to develop acute kidney injury (13.2% vs. 1.7%, p-value < 0.001), hypoxemia during hospitalization (63.7% vs. 36.2%, p-value <0.001), to be intubated within 30-days of admission (22% vs. 11.4%, p-value = 0.023) and to die within 60 days of admission (41.8% vs. 6.3%, p-value<0.001) than patients with normal troponin level (Table 3). The length of hospital stay was not significantly different between the two groups.

**Table 3:** Outcomes of COVID-19 hospitalized patients.

	**Non-Elevated Troponin**	**Elevated Troponin**	**p-value**
**n**	229	91	
**Acute Kidney Injury-no. (%)**	4 (1.7)	12 (13.2)	<0.001
**Length of Stay-days- (median [IQR** ^**a**^**])**	6.00 3, 10]	6.50 [3, 16]	0.118
**Hypoxemia During Hospitalization -no. (%)**	83 (36.2)	58 (63.7)	<0.001
**Intubation in 30-Days from Admission-no. (%)**	26 (11.4)	20 (22.0)	0.023
**Death in 60-Days from Admission-no. (%)**	12 (6.3)	33 (41.8)	<0.001

^**a**^ interquartile range.

**Fig 1:**
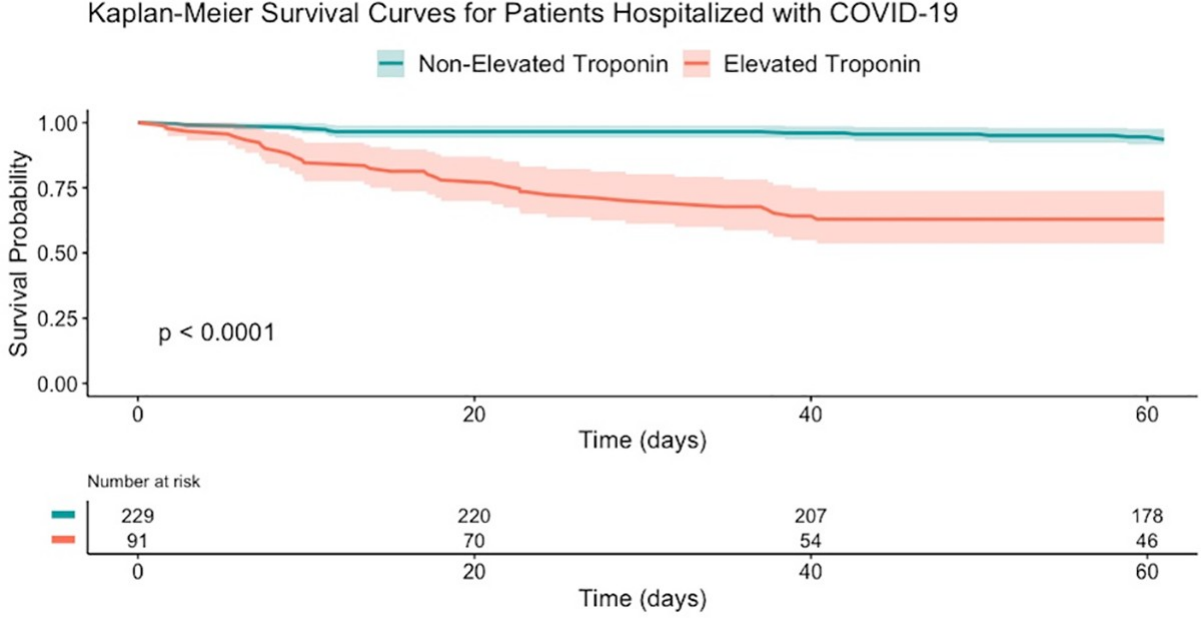
Kaplan-Meier survival curves for elevated and non-elevated troponin in patients hospitalized with COVID-19. Confidence band = 95%.

**Fig 2:**
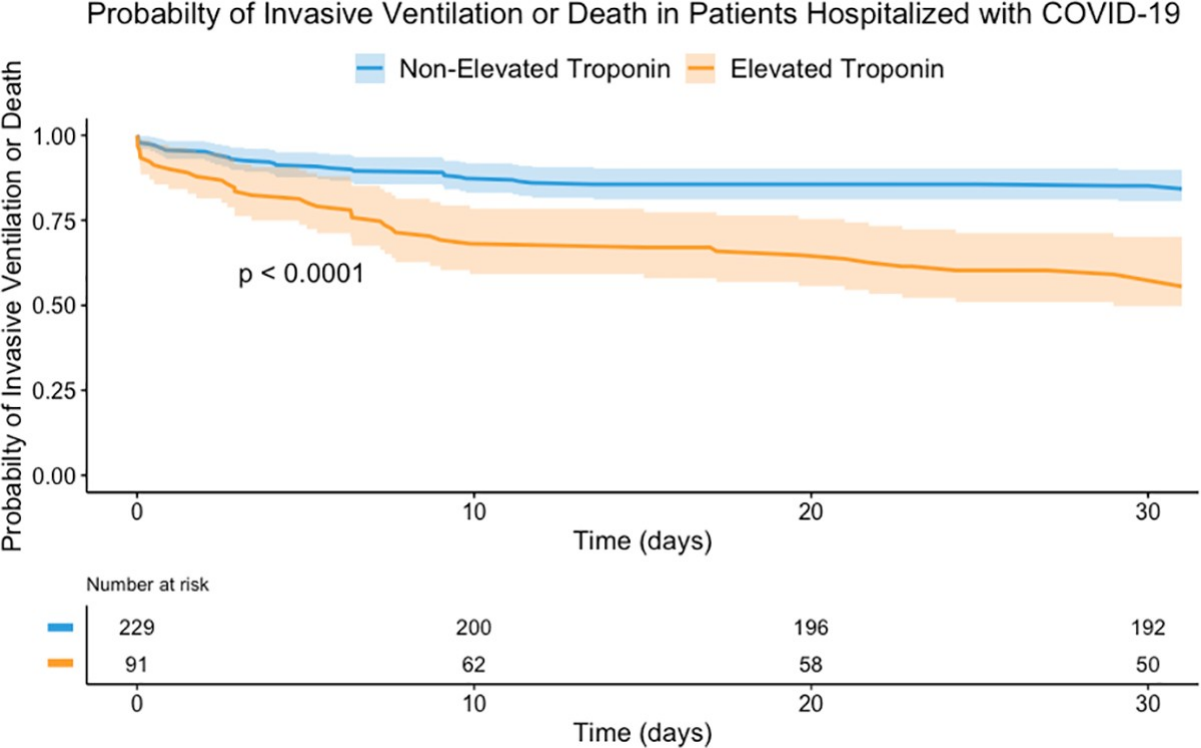
Cumulative incidence plot for a composite outcome of invasive ventilatory support and mortality. Confidence band = 95%; COVID-19, coronavirus disease 2019.

A Cox proportional hazards regression analysis for the same association, adjusted for sex, age, elevated troponin, comorbidities (atrial fibrillation, ischemic heart disease, heart failure, diabetes mellitus, chronic kidney disease, cerebrovascular accident, and hypertension), chronic medications (beta-blockers, angiotensin-converting-enzyme inhibitors [ACEI] or angiotensin II receptor blocker [ARBS], HMG CoA reductase inhibitors), low admission systolic blood pressure, admission creatinine level is presented in Table 4. The adjusted analysis shows a significantly increased risk of death within 60-days of admission, and a significantly increased risk of a composite outcome of invasive ventilation and 30-days mortality among patients diagnosed with COVID-19 and elevated troponin level as compared to non-elevated troponin level. A Kaplan-Meier plot stratified by troponin levels is shown in Fig 1. A cumulative incidence plot for the composite outcome of invasive ventilatory support and mortality is shown in Fig 2.

**Table 4:** Adjusted analysis for the association between troponin levels and 60-days mortality, and between troponin levels and 30-days composite of invasive ventilation support and mortality in patients hospitalized with COVID-19.

Characteristic	Mortality		Composite Outcome of Invasive Ventilation Support and Mortality	
	HR ^a^ [95% CI]	p-value	HR ^a^ [95% CI ^b^]	p-value
**Sex = Male**	2.00 [0.92, 4.36]	0.081	1.74 [0.98, 3.07]	0.058
**Age**	1.07 [1.04, 1.11]	<0.001	1.03 [1.01, 1.05]	0.001
**Elevated Troponin**	4.32 [2.08, 8.99]	<0.001	1.96 [1.15, 3.37]	0.014
**Atrial Fibrillation**	1.12 [0.52, 2.40]	0.781	1.09 [0.55, 2.13]	0.81
**Ischemic Heart Disease**	1.42 [0.65, 3.09]	0.383	0.90 [0.44, 1.82]	0.767
**Heart Failure**	1.03 [0.42, 2.55]	0.947	1.20 [0.55, 2.63]	0.647
**Diabetes Mellitus**	0.97 [0.45, 2.09]	0.941	1.13 [0.64, 1.98]	0.673
**Chronic Kidney Disease**	1.67 [0.69, 4.06]	0.258	0.76 [0.36, 1.61]	0.471
**Cerebrovascular Accident**	1.90 [0.83, 4.32]	0.128	1.33 [0.63, 2.82]	0.452
**Hypertension**	0.93 [0.42, 2.06]	0.863	0.69 [0.39, 1.25]	0.224
**Beta Blockers Therapy**	1.13 [0.56, 2.30]	0.731	1.31 [0.71, 2.39]	0.385
**ACE** ^**c**^ **or ARB** ^**d**^ **Therapy**	0.66 [0.23, 1.90]	0.442	0.70 [0.30, 1.64]	0.412
**HMG CoA Reductase Inhibitors**	1.13 [0.55, 2.29]	0.744	0.97 [0.56, 1.69]	0.918
**Low SBP** ^**e**^^,^ ^**f**^	2.38 [1.09, 5.21]	0.03	4.05 [2.26, 7.27]	<0.001
**Creatinine level** ^**f**^	1.02 [0.69, 1.52]	0.91	1.03 [0.77, 1.37]	0.857
**Lymphopenia** ^**f**^	0.65 [0.33, 1.29]	0.219	1.13 [0.68, 1.87]	0.631

^a^ hazard ratio.

^b^ confidence interval.

^c^ angiotensin-converting-enzyme inhibitors.

^d^ angiotensin II receptor blocker.

^e^ systolic blood pressure.

^f^ Measured within 24-hours from admission.

### Medical record inspection

Among 91 patients diagnosed with COVID-19 and elevated troponin, eight patients (8.79%) had newly significant ECG changes. Three of them had a low voltage of QRS amplitude, two had new-onset atrial fibrillation, and three had extensive ST elevation (STE). Of the three patients with STE, one was diagnosed with STE myocardial infarction (STEMI) a week before the COVID-19 diagnosis, and underwent percutaneous coronary intervention (PCI)- Therefore the ECG findings were regarded as reminder findings with no special treatment. The two other patients presented with STE on the ECG were immediately taken to coronary catheterization with a suspected STEMI. Of these, one had significant coronary stenosis requiring the placements of two drug-eluting stents, while the other was found to have normal coronaries. A detailed specification of ECG findings of the sub-group of deceased patients hospitalized with COVID-19 and elevated troponin can be found in S4 Table.

Two patients with COVID-19 and elevated troponin were diagnosed with pulmonary embolism based on a computed tomography angiography and doppler ultrasound of the lower extremities demonstrating deep vein thrombosis.

To further analyze the cause of death of the patients who were deceased and had elevated troponin, we reviewed their medical records for the cause of death (S4 Table). Out of 33 patients, in 30 patients (91%), the cause of death was respiratory failure following pulmonary complications of Coronavirus disease 2019 (COVID-19); 28 patients died from direct COVID-19 complications, whereas two died from suspected secondary bacterial pneumonia. In two patients the reason for death was undetermined and in one patient the cause of death was ascribed to foot ulcer complications with severe sepsis.

## Discussion

In this retrospective cohort study, we demonstrated factors that are associated with elevated troponin in patients with COVID-19, and showed that elevated troponin is associated with a poor prognosis in patients hospitalized with COVID-19. Moreover, its prognostic significance remains after adjustment for other variables. We showed that elevated troponin levels were prevalent among hospitalized COVID-19 patients (28.44% of tested patients) and were found to be associated with reduced survival as well as with an increased risk of a composite outcome of invasive ventilatory support and death. Elevated troponin was also associated with a higher risk for other in-hospital adverse events, such as acute kidney injury and low oxygen saturation. These findings strengthen the prognostic value of the myocardial injury, as measured by serum troponin, in patients hospitalized with COVID-19, as was shown in prior studies. A retrospective cohort conducted by Shi et al. [10], as well as recently published retrospective studies by Lala et al. [11] and Lombardi et al. [15] demonstrated that elevated troponin was associated with a significantly higher mortality rate and adverse in-hospital outcomes.

As described in prior studies, patients with elevated troponin levels were older and had higher rates of underlying comorbidities [2, 10, 11, 13–15]. Accordingly, they were more likely to be treated with chronic medicine such as beta-blockers, HMG-CoA-reductase inhibitors.

Low systolic blood pressure (<90mmHg) and increased admission creatinine levels were found to be associated with the development of cardiac injury in patients hospitalized with COVID-19. Female sex was shown to be a predictor for myocardial injury, but it may be explained by selection bias. Male patients were more likely to be tested for troponin levels, and possibly female patients were tested when being older or exhibiting more severe symptoms. When stratified by sex, there was a significant age difference between female patients with elevated troponin levels and males, with female patients being significantly older.

Several possible explanations were suggested for the mechanism by which COVID-19 causes myocardial injury [21, 22]. Increased frequency of pulmonary embolism, acute respiratory distress syndrome, in combination with a systemic inflammatory storm, are some of the theories explaining myocardial injury in COVID-19 patients [22]. The systemic inflammatory storm, in particular, can lead to ischemic myocardial injury in the context of a mismatch between oxygen supply and demand, also termed as type 2 myocardial infarction [19]. Local infection within the myocardium with non-clinical overt inflammation was also suggested [23, 24].

We assume that as in other medical conditions, the elevated troponin levels reflect myocardial injury as a part of the inflammatory state of the COVID-19 infection. As most patients in our study died from respiratory complications, we believe that the presence of myocardial injury signifies a more severe infection and inflammatory state, leading to a worse phenotype of its most common presentation, COVID-19 pneumonia. This assumption is supported by a recently published study by Metkus et al. [25] which demonstrated that COVID-19–mediated cardiac injury is less common in COVID-19 ARDS than in non-COVID ARDS after adjustment, concluding that its adverse prognosis relates largely to multisystem organ involvement and critical illness. Troponin elevation during the COVID-19 infection may also result from direct damage to the myocardium by the virus as well as represent right ventricular strain secondary to the acute pulmonary disease. These possible explanations are further supported by features of cardiac pathology in COVID-19 infection, which showed acute right ventricular dilatation in some of the examined hearts as an indicator of extreme stress secondary to acute pulmonary disease, and a direct myocyte damage by viral infection of the endothelial compartment [26].

Cardiac arrests and arrythmias were described in COVID-19 hospitalized patients in several studies and reports [16, 27]. These cardiac conditions, which present potential cause of death induced by myocardial injury, were associated with disease severity. Moreover, these conditions may also promote myocardial injury by themselves. However, after reviewing the medical records of COVID-19 patients with elevated troponin, including ECG tests, only a few had findings pointing to a specific etiology, accentuating its significance as a non-dependent prognostic marker.

To-date, this is the first study to evaluate ECG findings in a large population with COVID-19 infection and elevated troponin levels, comparing them to previous individual exams, and further tracking their coronary catheterization results. This in-depth analysis enabled us to demonstrate that troponin levels may be of prognostic significance even without any evidence for myocardial ischemia on ECG, with two patients demonstrated to have normal coronaries on coronary catheterization.

### Limitations

The small sample size in this study limits the interpretation of the results. Furthermore, this research was conducted in a single tertiary medical center. troponin levels were measured according to the discretion of the attending physician and, therefore, selection bias might have occurred. Indeed, it is evident that patients who had troponin measured were older and tended to suffer from more background diseases, including ischemic heart disease.

Acute kidney injury was defined according to the KDIGO definition of a ratio of more than 1.5 between the maximal and minimal creatinine levels at a seven day period. Urine output and 48-hours elevation were not considered, as well as changes in creatinine values that were non-minimal or non-maximal. Thus, there is an underestimation of AKI events.

Body mass index was not calculated routinely thus was not retrieved, as well as di-dimer and Creatine kinase, which were not routinely measured. Furthermore, echocardiography test, which may have added aetiologic and prognostic information was not performed during hospitalization to the studied population.

## Conclusions

This study demonstrates that myocardial injury, as indicated by elevated troponin levels, is an important prognostic factor for people diagnosed and hospitalized with COVID-19. Myocardial injury represents a poor prognosis and a higher risk for kidney function deterioration, oxygen support, invasive ventilation support, and mortality. This research emphasizes the importance of measuring troponin levels in all COVID-19 hospitalized patients, even without clinical evidence of myocardial injury. This will enable risk stratification, directing clinical decisions and intensity of care in the emergency room, and throughout hospitalization.

## Supporting information

S1 Table Normal laboratory reference range. (DOCX)

S2 Table Characteristics of patients at baseline. (DOCX)

S3 Table Characteristics at baseline of patients with elevated troponin levels according to sex. (DOCX)

S4 Table Electrocardiogram findings and cause of death of patients COVID-19 hospitalized patients with elevated troponin levels. (DOCX)

S1 Fig Troponin levels of patients hospitalized with COVID-19. (DOCX)

S2 Fig Elevated troponin levels in patients hospitalized with COVID-19. (DOCX)

S3 Fig Troponin levels of female patients hospitalized with COVID-19. (DOCX)

S4 Fig Elevated troponin levels in female patients hospitalized with COVID-19. (DOCX)

S5 Fig Troponin levels of male patients hospitalized with COVID-19. (DOCX)

S6 Fig Elevated troponin levels in male patients hospitalized with COVID-19. (DOCX)
