# Supplementary material for: Myocardial injury in hospitalized patients with COVID-19 infection—Risk factors and outcomes
Source: PLoS One. 2021 Feb 26;16(2):e0247800. doi: 10.1371/journal.pone.0247800 (PMC7909655; doi:10.1371/journal.pone.0247800)
Supplement: S1 Table — (DOCX) [file pone.0247800.s001.docx]

**S1 Table: Normal laboratory reference range**

| **Laboratory test** | **Normal reference range** |
| --- | --- |
| Troponin-I HS (ng/l) | Male <20  Female <12 |
| Hemoglobin (g/dl) | Male 13.5-17.5  Female 11.7-15.7 |
| Lymphocytes (K/microL) | 1-4.8 |
| Platelets (K/µl) | 130-440 |
| Creatinine (mg/dl) | Male 0.67-1.17  Female 0.51-0.95 |
| Albumin (g/dl) | 3.6-5.5 |
| CRP (mg/l) | 0-5 |
