# Supplementary material for: Myocardial injury in hospitalized patients with COVID-19 infection—Risk factors and outcomes
Source: PLoS One. 2021 Feb 26;16(2):e0247800. doi: 10.1371/journal.pone.0247800 (PMC7909655; doi:10.1371/journal.pone.0247800)
Supplement: S2 Table — (DOCX) [file pone.0247800.s002.docx]

**S2 Table: Characteristics of patients at baseline**

|  | **Troponin not Tested** | **Troponin Tested** | **p-value** | **Missing values (%)** |
| --- | --- | --- | --- | --- |
| **n** | 239 | 320 |  |  |
| **Sex = Male (%)** | 130 (54.4) | 205 (64.1) | 0.026 | 0 |
| **Age- years (median [IQR ^a^])** | 52.85 [36.48, 68.30] | 64.76 [51.75, 74.31] | <0.001 | 0 |
| **Atrial Fibrillation- no. (%)** | 14 (5.9) | 36 (11.2) | 0.039 | 0 |
| **Heart Failure- no. (%)** | 11 (4.6) | 27 (8.4) | 0.107 | 0 |
| **Ischemic Heart Disease- no. (%)** | 11 (4.6) | 43 (13.4) | 0.001 | 0 |
| **Chronic Kidney Disease- no. (%)** | 18 (7.5) | 39 (12.2) | 0.097 | 0 |
| **Hypertension- no. (%)** | 60 (25.1) | 138 (43.1) | <0.001 | 0 |
| **Cerebrovascular Accident- no. (%)** | 17 (7.1) | 29 (9.1) | 0.5 | 0 |
| **Chronic Obstructive Pulmonary Disease- no. (%)** | 6 (2.5) | 11 (3.4) | 0.702 | 0 |
| **Diabetes Mellitus- no. (%)** | 45 (18.8) | 83 (25.9) | 0.06 | 0 |
| **Dyslipidemia- no. (%)** | 42 (17.6) | 105 (32.8) | <0.001 | 0 |
| **Malignancy- no. (%)** | 18 (7.5) | 39 (12.2) | 0.097 | 0 |
| **ACEI ^b^ / ARB ^c^ Therapy- no. (%)** | 16 (6.7) | 36 (11.2) | 0.092 | 0 |
| **Beta-blockers Therapy- no. (%)** | 33 (13.8) | 81 (25.3) | 0.001 | 0 |
| **HMG CoA reductase inhibitors Therapy- no. (%)** | 39 (16.3) | 108 (33.8) | <0.001 | 0 |
| **Temperature- celsius (median [IQR ^a^])** | 37.40 [37.00, 38.20] | 38.00 [37.30, 38.62] | <0.001 | 0.2 |
| **SBP ^d, e^-** **mmHg^f^ (median [IQR ^a^])** | 111.00 [101.00, 122.00] | 109.00 [98.00, 122.00] | 0.198 | 0 |
| **Creatinine ^e^-** **mg/dl (median [IQR ^a^])** | 0.81 [0.64, 0.97] | 0.95 [0.74, 1.17] | <0.001 | 10 |
| **Albumin ^e^-g/dl (median [IQR ^a^])** | 3.90 [3.50, 4.35] | 3.70 [3.50, 4.10] | 0.004 | 13.4 |
| **Hemoglobin ^e^-g/dl (median [IQR ^a^])** | 13.30 [11.93, 14.36] | 13.39 [12.17, 14.43] | 0.446 | 10.2 |
| **Lymphocytes ^e^ K/µl (median [IQR ^a^])** | 1.17 [0.74, 1.62] | 1.00 [0.68, 1.39] | 0.012 | 10 |
| **Platelets ^e^-** **K/µl (median [IQR ^a^])** | 190.00 [148.50, 240.50] | 190.00 [144.00, 242.25] | 0.771 | 10 |
| **CRP ^e,^ ^g^-** **mg/l (median [IQR ^a^])** | 25.84 [5.66, 96.26] | 73.98 [27.00, 138.01] | <0.001 | 13.2 |
| ^a^ interquartile range  ^b^  angiotensin-converting-enzyme inhibitors  ^c^ angiotensin II receptor blocker  ^d^ systolic blood pressure  ^e^ Measured within 24-hours from admission  ^f^  millimeter of mercury  ^g^ c-reactive protein | | | | |
