# Supplementary material for: Myocardial injury in hospitalized patients with COVID-19 infection—Risk factors and outcomes
Source: PLoS One. 2021 Feb 26;16(2):e0247800. doi: 10.1371/journal.pone.0247800 (PMC7909655; doi:10.1371/journal.pone.0247800)
Supplement: S3 Table — (DOCX) [file pone.0247800.s003.docx]

**S3 Table: Characteristics at baseline of patients with elevated troponin levels according to sex**

|  | Females | Males | P-value |
| --- | --- | --- | --- |
| n | 41 | 50 |  |
| Age- years (median [IQR a]) | 79.04 [66.87, 87.26] | 69.51 [60.48, 75.97] | 0.008 |
| Atrial fibrillation- no. (%) | 9 (22.0) | 10 (20.0) | 1 |
| Heart failure- no. (%) | 6 (14.6) | 11 (22.0) | 0.531 |
| Malignancy- no. (%) | 7 (17.1) | 8 (16.0) | 1 |
| Ischemic heart disease- no. (%) | 6 (14.6) | 16 (32.0) | 0.093 |
| Chronic Obstructive Pulmonary Disease- no. (%) | 4 (9.8) | 3 (6.0) | 0.784 |
| Diabetes mellitus- no. (%) | 8 (19.5) | 22 (44.0) | 0.025 |
| Hypertension- no. (%) | 25 (61.0) | 29 (58.0) | 0.942 |
| Hyperlipidemia- no. (%) | 15 (36.6) | 19 (38.0) | 1 |
| Chronic kidney disease- no. (%) | 5 (12.2) | 15 (30.0) | 0.074 |
| Cerebrovascular Accident - no. (%) | 5 (12.2) | 7 (14.0) | 1 |
| ACEI b / ARB c Therapy- no. (%) | 6 (14.6) | 9 (18.0) | 0.883 |
| Beta-blockers Therapy- no. (%) | 18 (43.9) | 21 (42.0) | 1 |
| HMG CoA reductase inhibitors Therapy- no. (%) | 17 (41.5) | 23 (46.0) | 0.825 |

a interquartile range

b angiotensin-converting-enzyme inhibitors

c angiotensin II receptor blocker
