# Supplementary material for: Myocardial injury in hospitalized patients with COVID-19 infection—Risk factors and outcomes
Source: PLoS One. 2021 Feb 26;16(2):e0247800. doi: 10.1371/journal.pone.0247800 (PMC7909655; doi:10.1371/journal.pone.0247800)
Supplement: S4 Table — (DOCX) [file pone.0247800.s004.docx]

**S4 Table: Electrocardiogram findings and cause of death of patients** **COVID-19 hospitalized patients with elevated troponin levels**

| **age of death** | **Cause of death** | **ECG^a,b^ findings** |
| --- | --- | --- |
| 47 | COVID-19 with respiratory failure | sinus tachycardia |
| 49 | COVID-19 with respiratory failure | sinus tachycardia |
| 50 | COVID-19 with respiratory failure | ST elevations (inferior leads, V5-6)^c^ |
| 62 | foot ulcer complications with severe sepsis | No significant changes were found |
| 62 | COVID-19 with respiratory failure | sinus tachycardia |
| 65 | COVID-19 with respiratory failure | No significant changes were found |
| 68 | COVID-19 with respiratory failure | No significant changes were found |
| 70 | COVID-19 with respiratory failure | No significant changes were found |
| 71 | COVID-19 with respiratory failure | No significant changes were found |
| 73 | COVID-19 with respiratory failure | No significant changes were found |
| 73 | COVID-19 with respiratory failure | No significant changes were found |
| 73 | COVID-19 with respiratory failure | No significant changes were found |
| 74 | COVID-19 with respiratory failure | No significant changes were found |
| 76 | COVID-19 with respiratory failure | No significant changes were found |
| 76 | COVID-19 with respiratory failure | No significant changes were found |
| 77 | Undetermined | No significant changes were found |
| 81 | COVID-19 with respiratory failure | No significant changes were found |
| 82 | Secondary bacterial pneumonia following COVID-19 | No significant changes were found |
| 82 | COVID-19 with respiratory failure | No significant changes were found |
| 82 | COVID-19 with respiratory failure | sinus tachycardia |
| 83 | COVID-19 with respiratory failure | No significant changes were found |
| 83 | COVID-19 with respiratory failure | sinus tachycardia |
| 85 | COVID-19 with respiratory failure | No significant changes were found |
| 85 | COVID-19 with respiratory failure | No significant changes were found |
| 87 | COVID-19 with respiratory failure | No significant changes were found |
| 88 | COVID-19 with respiratory failure | No significant changes were found |
| 89 | Undetermined | No significant changes were found |
| 90 | COVID-19 with respiratory failure | No significant changes were found |
| 90 | COVID-19 with respiratory failure | No significant changes were found |
| 90 | COVID-19 with respiratory failure | No significant changes were found |
| 93 | COVID-19 with respiratory failure | No significant changes were found |
| 94 | Secondary bacterial pneumonia following COVID-19 | No significant changes were found |
| 95 | COVID-19 with respiratory failure | No significant changes were found |

^a^ electrocardiogram

^b^ Compared to baseline electrocardiogram if available

^c^ normal coronaries on cardiac catheterization
