# Supplementary figures and images for: Myocardial injury in hospitalized patients with COVID-19 infection—Risk factors and outcomes
Source: PLoS One. 2021 Feb 26;16(2):e0247800. doi: 10.1371/journal.pone.0247800 (PMC7909655; doi:10.1371/journal.pone.0247800)

**S1 Fig. Troponin levels of patients hospitalized with COVID-19**


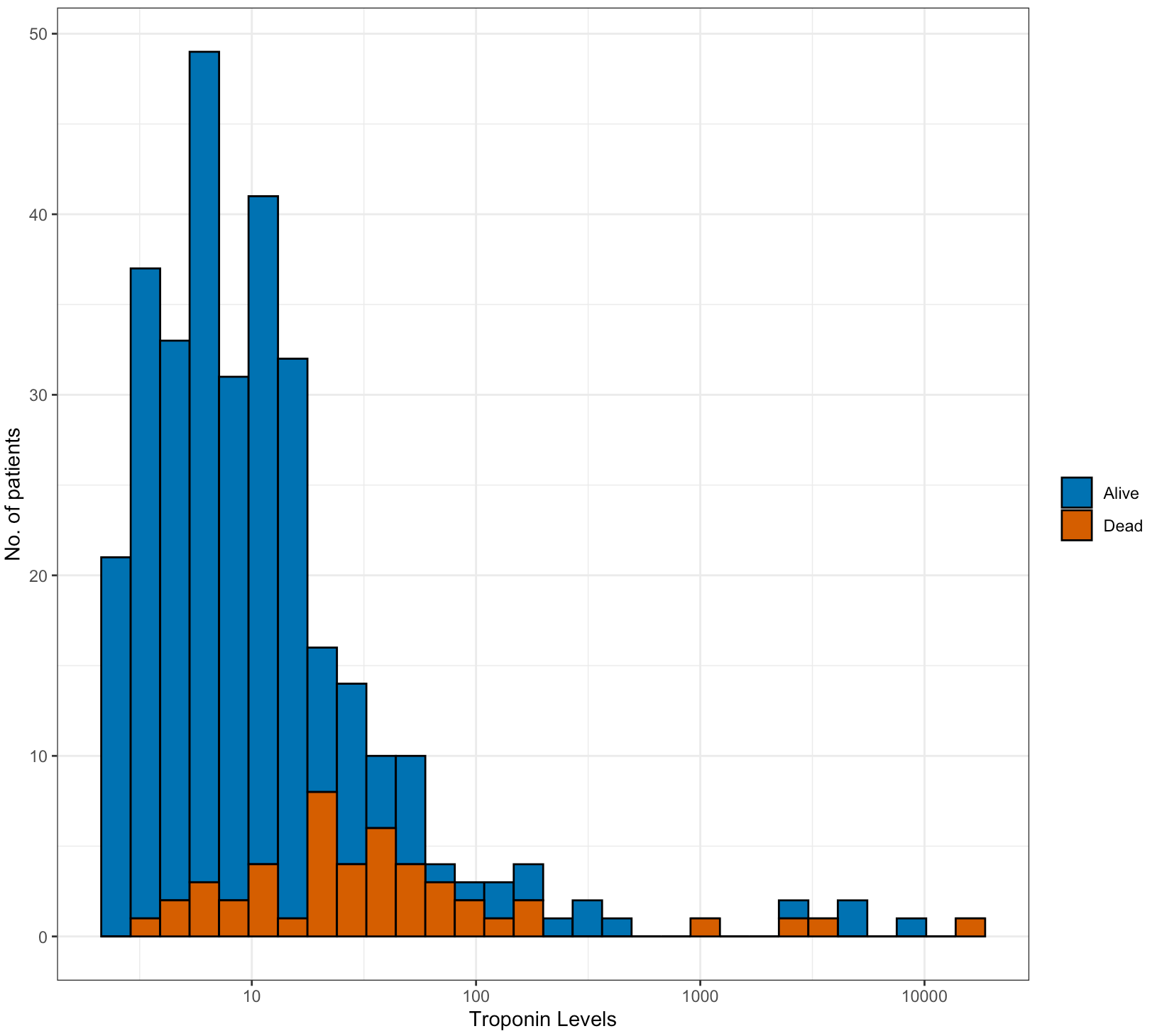

Supplement: S1 Fig — (DOCX) [file pone.0247800.s005.docx]

**S2 Fig. Elevated troponin levels in patients hospitalized with COVID-19**


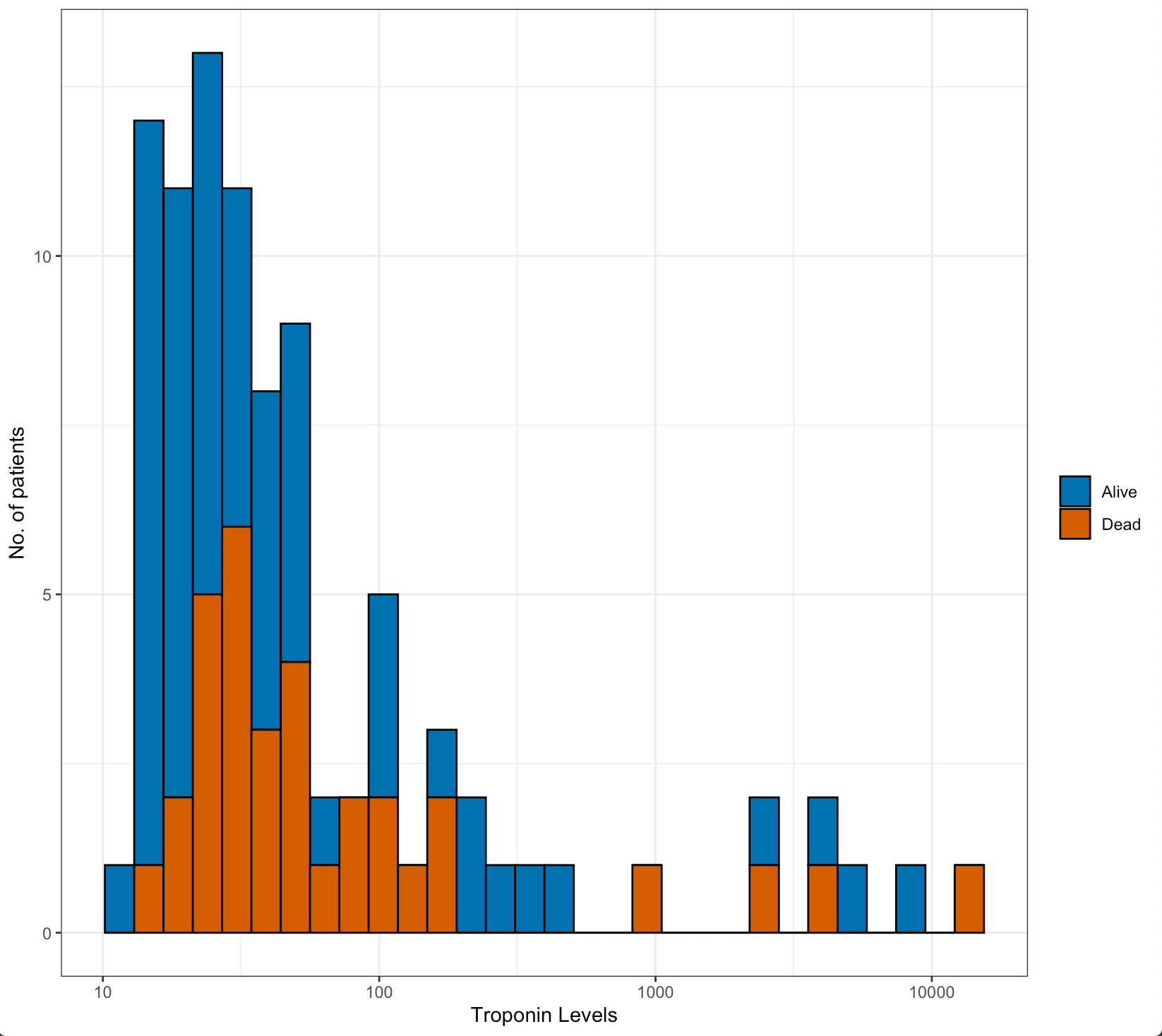

Supplement: S2 Fig — (DOCX) [file pone.0247800.s006.docx]

**S3 Fig. Troponin levels of female patients hospitalized with COVID-19**


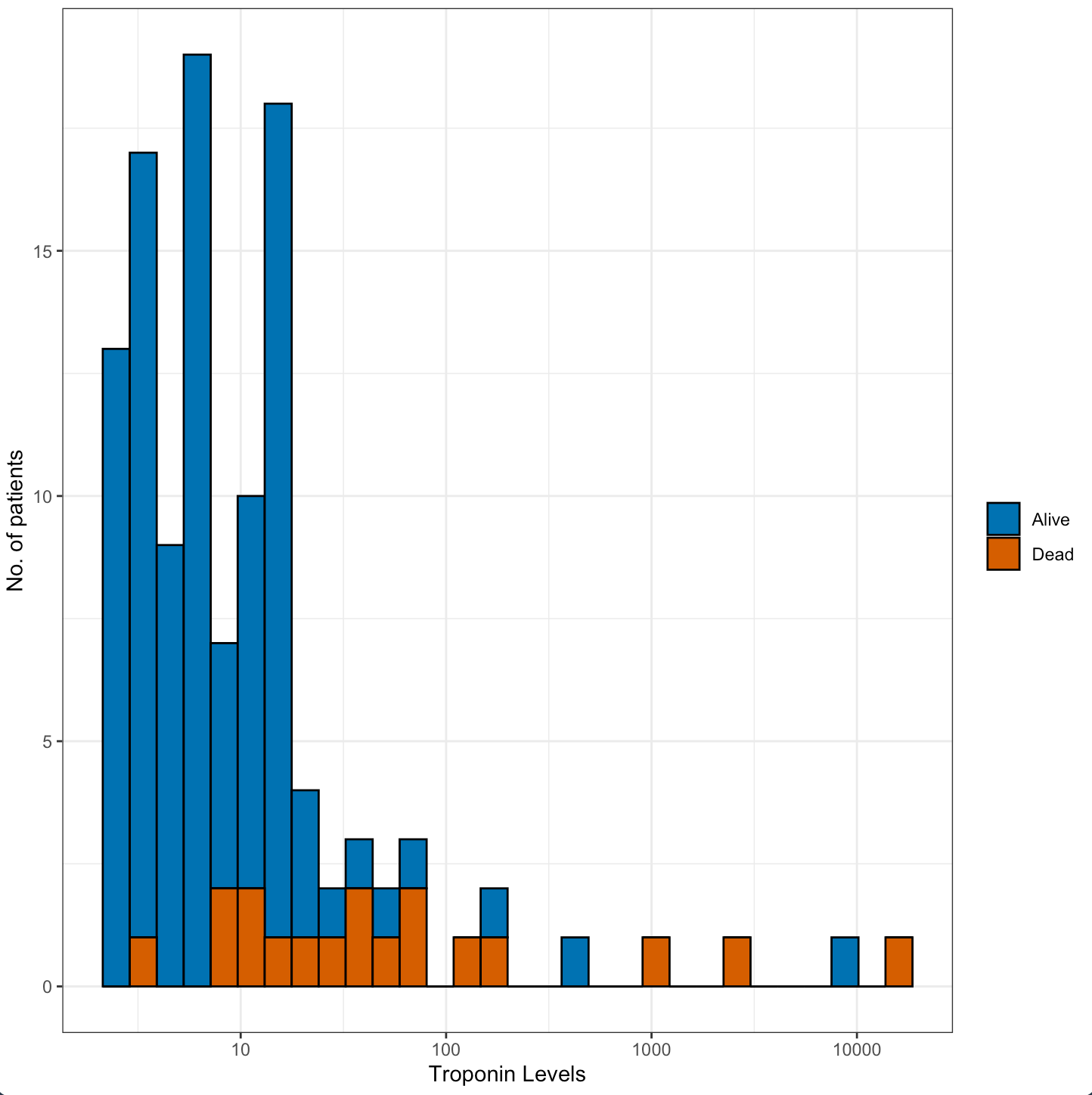

Supplement: S3 Fig — (DOCX) [file pone.0247800.s007.docx]

**S4 Fig. Elevated troponin levels in female patients hospitalized with COVID-19**


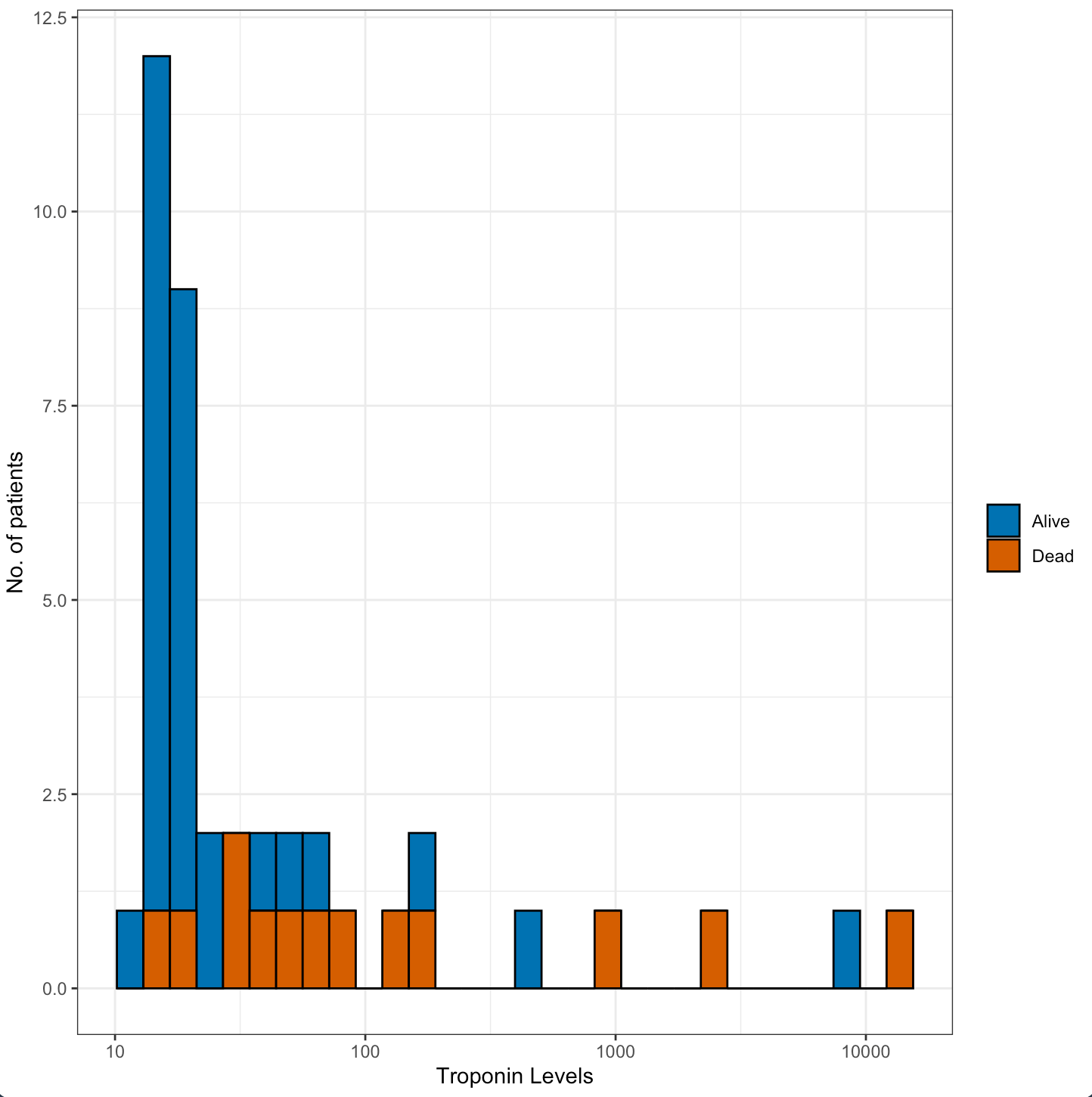

Supplement: S4 Fig — (DOCX) [file pone.0247800.s008.docx]

**S5 Fig. Troponin levels of male patients hospitalized with COVID-19**


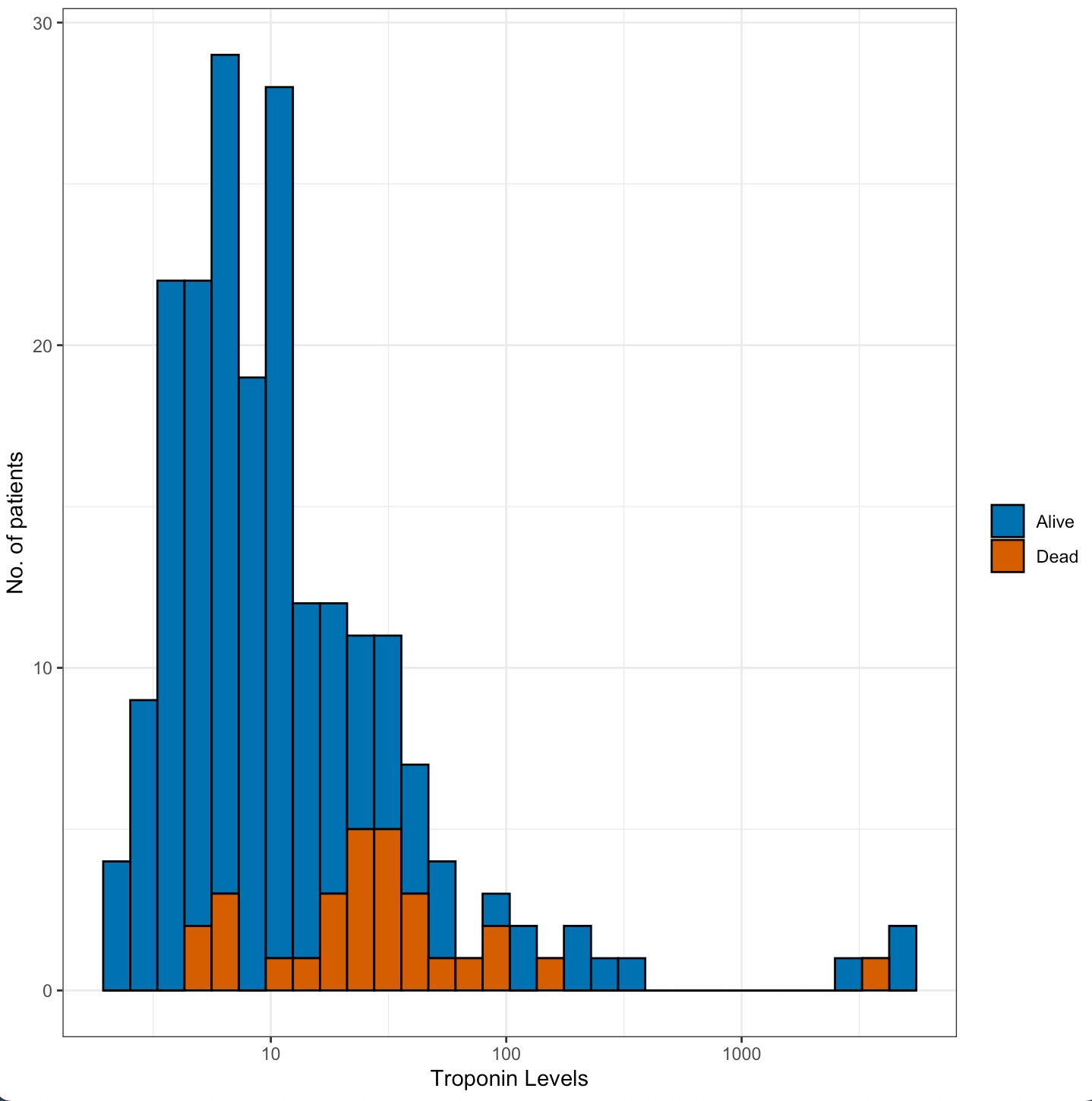

Supplement: S5 Fig — (DOCX) [file pone.0247800.s009.docx]

**S6 Fig. Elevated troponin levels in male patients hospitalized with COVID-19**


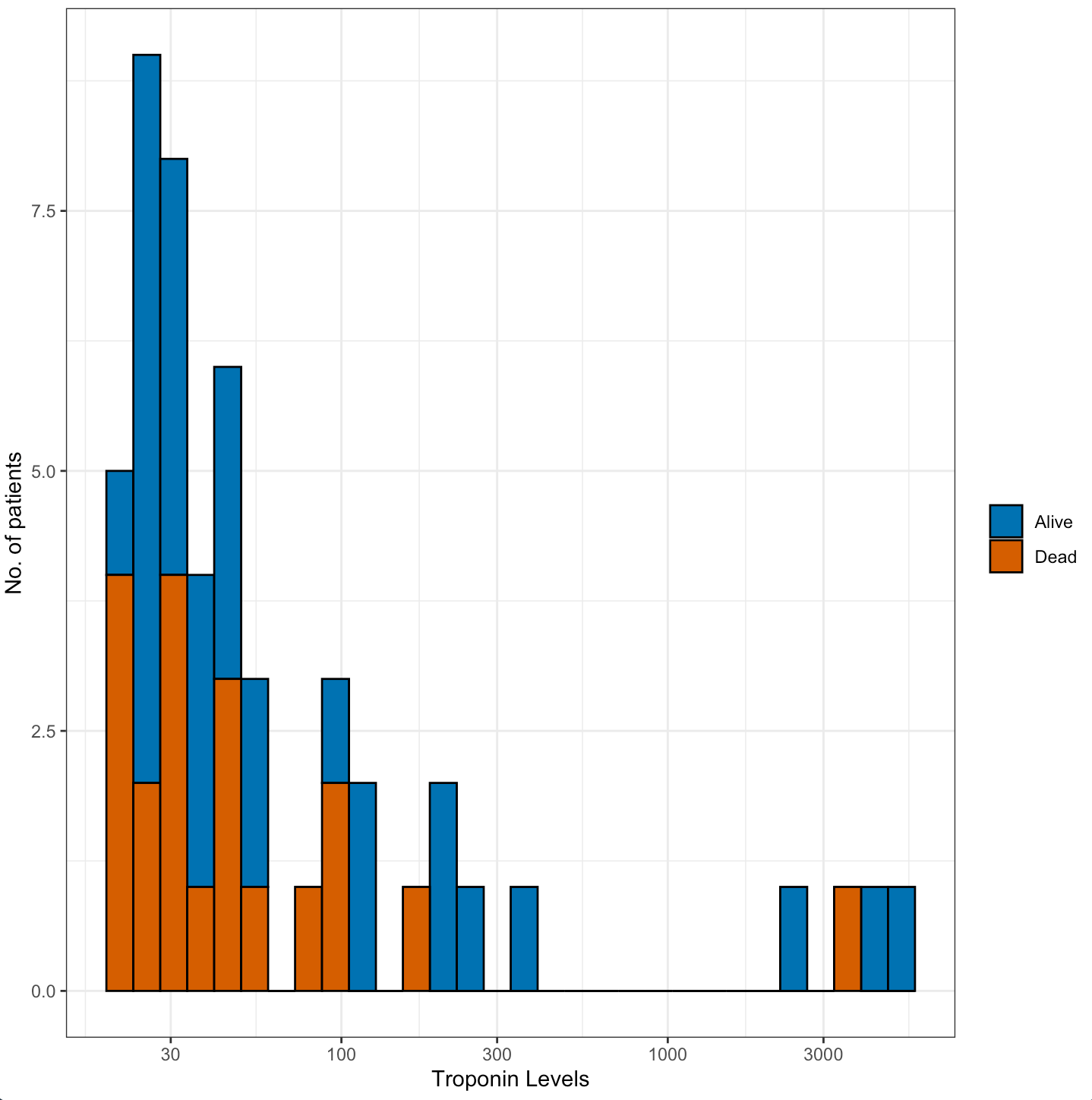

Supplement: S6 Fig — (DOCX) [file pone.0247800.s010.docx]
